# EMERGING BIOMARKERS OF MITOCHONDRIAL HEALTH

**DOI:** 10.1093/geroni/igaf122.1667a

**Published:** 2025-12-31

**Authors:** Anthony Molina

**Affiliations:** University of California, San Diego, La Jolla, California, United States

## Abstract

Advances in human mitochondrial bioenergetic profiling have improved our understanding of how cellular metabolism can drive a wide array of age-related conditions, including changes in physical and cognitive performance with advancing age. This presentation will review new techniques and approaches that we are applying in clinical studies to better understand age-related bioenergetic decline and its consequences. As mitochondrial therapeutics are increasingly recognized to have potential benefits for promoting healthy longevity, biomarkers of mitochondrial health have an important role in clinical trials evaluating promising gerotherapeutic interventions.

